# King Edward's Vaccine Treatment

**Published:** 1910-05-28

**Authors:** 


A Journal of

The Medical Sciences and Hospital Administration.

New Series. No. 171, Vol. VII. [No. 1243, Vol. XLVIII.] SATURDAY, MAY 28, 1910.

### King Edward's Vaccine Treatment.

Seldom, if ever, have the anti-vivisection fanatics been betrayed by their ignorance and their intemperance into a falser position than that which they have just been forced to evacuate by a message wrung from Queen Alexandra. While Edward VII. lay still unburied, some of these incomprehensible zealots distributed broadcast in the streets of his capital—and, for aught we know, probably all through his kingdom—a leaflet suggesting that the cause of his Majesty's death was a course of vaccine treatment which he had undergone. To such an extent has this scandalous lie been circulated that the widowed Queen could no longer bear to treat it with the silent contempt which is usually the most appropriate comment on the fantastic untruths of the anti-vivisector. The *Court Circular* contains a notification that the report has caused great grief to the Queen-Mother, that before the journey to Biarritz the late King had never felt better in health and spirits, and that the vaccine treatment had kept his Majesty in excellent health, to his own entire satisfaction, for fifteen months. Queen Alexandra further wishes it to be stated that his Majesty's attack at Biarritz was in no way related to his previous course of treatment. That this official pronouncement represents the truth, the whole truth, and nothing but the truth, is, it is no breach of confidence to assert, common knowledge in medical circles. As can be affirmed without any fear of contradiction, about the beginning of last year his Majesty's advisers felt that a continuation of the recurring catarrhal and influenzal attacks from which he had been suffering would be extremely dangerous, and they accordingly advised that a course of preventive vaccine therapy should be carried out. After accepting this advice and undergoing the treatment, the late King remained entirely free from all trouble of that sort until the final breakdown, and he himself was highly pleased with the unequivocal success of the vaccines used. Simultaneously with the publication of this dignified but severe rebuke to these busybodies and disseminators of untruth, we are glad to notice the repudiation of the leaflet by Lord Llangattock and Mr. Stephen Coleridge on behalf of the National Anti-Vivisection Society. But we cannot entirely refrain from noticing that these gentlemen believe that "the dissemination of this leaflet is calculated to misrepresent and gravely injure the cause for which we labour"; it is not stated in their letter that they disbelieve the absurd story contained in it, though probably at the time their letter was written they had not seen the *Court Circular* to which we have referred. Lord Llangattock and Mr. Coleridge may reasonably be reminded that the frame of mind of those who did actually circulate the leaflet is absolutely typical of the members of the Anti-Vivisection Society. Acts as ill-advised and statements as untrue are nothing new in the propaganda of these amazing societies, though it is not often that the searchlight of truth is directed upon them with such effect as in the present case.

